# Stories of change in nutrition from Africa and Asia: an introduction to a special series in Food Security

**DOI:** 10.1007/s12571-021-01166-8

**Published:** 2021-05-06

**Authors:** Stuart Gillespie, Jody Harris, Nicholas Nisbett, Mara van den Bold

**Affiliations:** 1 International Food Policy Research Institute, Washington, D.C., USA; 2 Institute of Development Studies, Brighton, UK; 3 World Vegetable Center, Bangkok, Thailand

**Keywords:** Nutrition, Policy, System change, Africa, Asia

## Abstract

Malnutrition in all its forms continues to be a massive global challenge, and the past decade has seen a growing political attention to addressing malnutrition in different contexts. What has been largely missing so far, and is in growing demand from countries, is tangible, practical and rigorous insights and lessons (from other countries or contexts) on how to translate this burgeoning political momentum into effective policies and programme implementation strategies – and ultimately impact on the ground. This new climate of learning from experience and evidence led to the launch in 2015 of the Stories of Change initiative. This series presents a second wave of studies from six countries (Tanzania, Rwanda, Vietnam, Ghana, Burkina Faso, Nigeria,) and three Indian states (Chhattisgarh, Gujarat, Tamil Nadu). These provide clear evidence combined with compelling narratives on what drives success in addressing all forms of malnutrition – evidence that is necessary for turning global momentum into actual results on the ground. This introductory Opinion is published with the first set of papers. It will be followed by a thorough synthesis of papers as a conclusion of the Series. We hope that the lessons embedded in these Stories of Change will inform and inspire the deliberations and outcomes of the UN Food Systems Summit and the second Nutrition for Growth Summit to be held this year, and the actions of those in the global food and nutrition system working for positive change.

## Stories of Change: A history and rationale for experiential learning

Malnutrition continues to be a massive global challenge. One in nine people in the world is hungry, and one in three is overweight or obese, according to the Global Nutrition Report (Development Initiatives 2020). These figures pre-date the COVID-19 pandemic, and the situation is very likely to have worsened during 2020 (Headey et al. 2020, Savary et al. 2020). While the World Health Assembly has endorsed ten global nutrition targets for 2025, not one single country is on track to meet all of them, and just 8 of 194 countries are on track to meet four targets (Development Initiatives 2020): Almost a quarter of all children in the world under 5 years of age are stunted. At the same time, overweight and obesity prevalence increases unabated, rapidly, in nearly every country in the world. An increasing number of countries in the world experience the *double burden of malnutrition*, where undernutrition coexists with overweight, obesity and other diet-related non-communicable diseases (NCDs). Africa and Asia collectively share the largest numbers of all forms of malnutrition.

The past decade has seen a growing political attention to addressing malnutrition in different contexts. This is illustrated by multiple initiatives, events and publications.^1^ What has been largely missing so far, and is in growing demand from countries, is tangible, practical and rigorous insights and lessons (from other countries or contexts) on how to translate this burgeoning political momentum into effective policies and programme implementation strategies – and ultimately impact on the ground.

This new climate of learning from experience and evidence led to the launch in 2015 of the *Stories of Change* initiative. Six case studies were undertaken – in Senegal, Ethiopia, Zambia, Nepal, Bangladesh, and Odisha, India (Cunningham et al. 2017, Harris et al. 2017, Kampman et al. 2017, Kohli et al. 2017, Nisbett et al. 2017, Warren and Frongillo 2017). These studies focused on examining changes in outcomes and drivers of child stunting (Headey et al. 2017), changes in policy and practice that were likely to have shaped them over time (Gillespie et al. 2017) and community experiences of these changes (Nisbett et al. 2017). Importantly, in a context in which the focus is increasingly on ‘evidence hierarchies’ of different forms of quantitative evidence, they also focused on capturing *experiential learning* – from policy makers, implementers, and nutrition leaders – on how to address malnutrition challenges in different contexts (Gillespie et al. 2016).

All case studies use a mixed methods approach, triangulating different forms of quantitative and qualitative data collection and analysis to examine nutrition-relevant change over time, usually the previous 15–20 years. These include analyses of changes in nutrition outcomes as well as identification of underlying food, health and care drivers of these changes where data allowed, through decomposition analyses quantifying contribution to change. These assessments are complemented with analysis of policy processes, to understand how and why different policy decisions were taken and what the implications of these decisions were for malnutrition and its identified drivers. These studies use theory-driven desk reviews of nutrition-relevant policies and programs, stakeholder mapping to identify policy networks and actors, and interviews with key stakeholders in various sectors and at different administrative levels to understand actor perspectives and actions. Policy process studies are guided by various conceptual frameworks – generally focused on examining changes in political commitment, policy coherence (horizontal and vertical), programme implementation – and apply social and political science theory in analysing change. The combination of these very different analyses is powerful in providing different perspectives on understanding change.

From its inception, the Stories of Change process revolved around a thorough and systematic stakeholder engagement process. This included in-country consultations as well as building on connections made through the research interviews and focus groups, in order to bring key nutrition policy and program actors into the studies -- from research design to dissemination of core lessons and recommendations. There were several audiences, from high-level champions to mid-level program implementers, public and private sector actors, civil society and academia. All emerging ‘draft stories’ were discussed by participants in national consultations before being finalized and published in different forms for different audiences. The ‘stories’ approach has also influenced other initiatives including the Scaling Up Nutrition (SUN) Movement, and Exemplars in Global Health which has used a similar approach to unpack the drivers of success in reducing child stunting rates in other countries, including Kyrgyz Republic and Peru (Heidkamp et al. 2021).

A synthesis of the initial ‘first wave’ studies reinforced the importance of a judicious mix of direct and indirect actions by several sectors. Key ingredients of positive change in most contexts included political commitment, horizontal and vertical policy coherence, accountability, data, leadership, capacity and finance (Gillespie et al. 2017). Such factors and processes needed to be present over time for progress to be made and sustained—but they will not look the same in every place. The choice of actual policy and programme actions will be driven by context—including the type of nutrition problem, available solutions, and the capacity to act. But overall, these were the foundational preconditions and drivers of change in undernutrition.

## Stories of challenge: Looking forward to nutrition this decade

*In December 2019, The Lancet* published a new series of papers on The Double Burden of Malnutrition (Horton 2019). A key paper in this series argued for a concerted focus on the design and implementation of *double duty actions* that had potential for simultaneously tackling undernutrition and overweight/obesity as the key feature of malnutrition in the twenty-first century (Hawkes et al. 2020). Nine of the ten recommended actions relate to redesigning and scaling up priority interventions and programs in health, social protection, education, and agri-food systems. The 10th focuses on implementation of policies to improve food environments. All these are important, but there is no upstream perspective on how political attention and policy traction is achieved (or not) in countries where different forms of malnutrition are prominent.

To start to fill this knowledge gap, between 2017 and 21, a second wave of studies was initiated in six additional countries, and three Indian states—Tanzania, Rwanda, Vietnam, Ghana, Burkina Faso, Nigeria, and India (Chhattisgarh, Gujarat, Tamil Nadu). Within this new wave of case studies, the focus has broadened from solely undernutrition to malnutrition in all its forms. Old challenges remain, which is why some studies concentrate on forms of malnutrition such as stunting and anaemia, documenting progress and identifying continued barriers; while others address the virtual absence of large-scale evidence-based stories on reducing overweight/obesity. For this reason, some of these case studies consider ‘stories of change’ looking back at efforts to address undernutrition, and others consider ‘stories of challenge’ looking forward to issues of overweight, obesity and the double burden. Change and challenge are similar words, and like crisis and opportunity, they’re flip sides of the same coin.

This new wave of studies will be published in *Food Security* journal as a Special Series throughout 2021. Stories of Change cover drivers of stunting reduction in Nigeria from 2003 to 2018 and in stunting and anaemia in Ghana between 2009 and 2018; changes in the enabling policy and political environment for nutrition in Nigeria and Rwanda; comparisons of stunting reduction in four Indian states; and how equity issues have shaped stunting reduction in Vietnam. Stories of Challenge cover urbanization and diet in Ghana; drivers of the double burden in South Africa; and food system drivers of dietary change in Vietnam and Zambia. Taken together with past work, the articles in this *Food Security* Special Series comprise the most comprehensive set of analyses of nutrition-relevant change and challenge ever undertaken. Insights and lessons – from a diversity of contexts, from a variety of viewpoints, and on a range of nutrition outcomes – can inform and start to improve agenda setting, design, implementation and scaling of relevant policies and programmes to address malnutrition in all its forms.

We need stories of change that are rigorous and coherent, that resonate and can catalyse change themselves. We need to continue to build and update a library of evidence and experience and ensure it is made accessible—and we need to become better storytellers, to get messages out to wider and more diverse audiences. Clear evidence combined with compelling narratives on what drives success in addressing all forms of malnutrition is necessary for turning global momentum into actual results on the ground.

This introductory Opinion is published with the first set of papers. It will be followed by a thorough synthesis of papers as a conclusion of the Series. We hope that the lessons embedded in these Stories of Change and Challenge will inform and inspire the deliberations and outcomes of the UN Food Systems Summit and the second Nutrition for Growth Summit to be held this year, and the actions of those in the global food and nutrition system working for positive change.

## Footnotes

1 Including the Scaling Up Nutrition (SUN) Movement in 2010, the Lancet Maternal and Child Nutrition Series in 2008 and 2013, six nutrition targets set at the 2012 World Health Assembly, the 2013 Nutrition for Growth (N4G) summit, which included pledges totaling USD23 billion for nutrition-related action, six Global Nutrition Reports (2014–20), Sustainable Development Goals (SDGs) that include a target to end all forms of malnutrition by 2030, and the UN Decade of Action on Nutrition (2016–25).

## References

[CR1] Cunningham K, Headey D, Singh A, Karmacharya C, Rana PP (2017). Maternal and child nutrition in Nepal: Examining drivers of progress from the mid-1990s to 2010s. Global Food Security.

[CR2] Initiatives D (2020). 2020 global nutrition report: Action on equity to end malnutrition.

[CR3] Gillespie S, Hodge J, Yosef S, Pandya-Lorch R (2016). Nourishing millions: Stories of change in nutrition.

[CR4] Gillespie S, van den Bold M, S. o. C. S. Team (2017). Stories of change in nutrition: An overview. Global Food Security.

[CR5] Harris J, Drimie S, Roopnaraine T, Covic N (2017). From coherence towards commitment: Changes and challenges in Zambia’s nutrition policy environment. Global Food Security.

[CR6] Hawkes C, Ruel MT, Salm L, Sinclair B, Branca F (2020). Double-duty actions: Seizing programme and policy opportunities to address malnutrition in all its forms. The Lancet.

[CR7] Headey D, Heidkamp R, Osendarp S, Ruel M, Scott N, Black R, Shekar M, Bouis H, Flory A, Haddad L (2020). Impacts of COVID-19 on childhood malnutrition and nutrition-related mortality. The Lancet.

[CR8] Headey D, Hoddinott J, Park S (2017). Accounting for nutritional changes in six success stories: A regression-decomposition approach. Global Food Security.

[CR9] Heidkamp, R. A., Piwoz, E., Gillespie, S., Keats, E. C., D’Alimonte, M. R., Menon, P., et al. (2021). Mobilising evidence, data, and resources to achieve global maternal and child undernutrition targets and the sustainable development goals: An agenda for action. *The Lancet,**397*(10282), 1400–1418. 10.1016/S0140-6736(21)00568-7 33691095

[CR10] Horton R (2019). A future direction for tackling malnutrition. Lancet.

[CR11] Kampman H, Zongrone A, Rawat R, Becquey E (2017). How Senegal created an enabling environment for nutrition: A story of change. Global Food Security.

[CR12] Kohli N, Avula R, van den Bold M, Becker E, Nisbett N, Haddad L, Menon P (2017). Reprint of what will it take to accelerate improvements in nutrition outcomes in Odisha? Learning from the past. Global Food Security.

[CR13] Nisbett N, Davis P, Yosef S, Akhtar N (2017). Bangladesh’s story of change in nutrition: Strong improvements in basic and underlying determinants with an unfinished agenda for direct community level support’. Global Food Security.

[CR14] Nisbett N, van den Bold M, Gillespie S, Menon P, Davis P, Roopnaraine T, Kampman H, Kohli N, Singh A, Warren A (2017). Community-level perceptions of drivers of change in nutrition: Evidence from South Asia and sub-Saharan Africa. Global Food Security.

[CR15] Savary S, Akter S, Almekinders C, Harris J, Korsten L, Rötter R, Waddington S, Watson D (2020). Mapping disruption and resilience mechanisms in food systems. Food Security.

[CR16] Warren AM, Frongillo EA (2017). Mid-level actors and their operating environments for implementing nutrition-sensitive programming in Ethiopia. Global Food Security.

